# Development of a 24-hour movement behaviors questionnaire (24HMBQ) for Chinese college students: validity and reliability testing

**DOI:** 10.1186/s12889-023-15393-5

**Published:** 2023-04-24

**Authors:** Jiaxin Zheng, Teck Cheng Tan, Kefeng Zheng, Tao Huang

**Affiliations:** Department of Physical Education, Shanghai Jiao Tong University, Shanghai, China

**Keywords:** 24-hour movement behaviors, Questionnaire, Reliability, Validity, College students

## Abstract

**Background:**

Physical activity (PA), sedentary behaviors (SB), and sleep are interrelated behavior components of a 24-hour day. Research interests continue to increase in examining the inter-relationship of three behaviors and their combined effects on health. The purpose of this study was to develop a comprehensive instrument to measure 24-hour movement behaviors for Chinese college students.

**Methods:**

The 24-hour movement behaviors questionnaire (24HMBQ) was developed based on a literature review and expert review. The target population (Chinese college students) and an expert panel assessed the face and content validity. After the final revision of the questionnaire, the participants (n = 229) were asked to complete the 24HMBQ twice to examine test-retest reliability. Convergent validity was evaluated using Spearman’s rho, by comparing the 24HMBQ estimates of sleep, SB, and PA with results derived from the Pittsburgh Sleep Quality Index (PSQI), the Adult Sedentary Behaviors Questionnaire in China (ASBQC), and the International Physical Activity Questionnaire - Short Form (IPAQ-SF).

**Results:**

The 24HMBQ exhibited good face validity and high acceptability to respondents. Regarding content validity, the S-CVI/UA and S-CVI/Ave were 0.88 and 0.97, respectively. As indicated by ICC, the test-retest reliability was considered moderate to excellent, ranging from 0.68 to 0.97 (P < 0.01). Regarding the convergent validity, correlations were 0.32 for the duration of sleep per day, 0.33 for total time of physical activity per day, and 0.43 for the duration of sedentary behaviors per day.

**Conclusion:**

The 24HMBQ is a feasible questionnaire with suitable validity and moderate to excellent test-retest reliability of all items. It is a promising tool to investigate 24-hour movement behaviors of Chinese college students. The 24HMBQ can be administrated in epidemiological studies.

**Supplementary Information:**

The online version contains supplementary material available at 10.1186/s12889-023-15393-5.

## Background

Over the past several decades, mounting evidence has suggested that high levels of physical activity (PA), low levels of sedentary behaviors (SB), and optimal sleep duration and quality are independently associated with various health benefits in all age groups [1–6]. PA, SB, and sleep are co-dependent since they are distributed within the 24-hour cycle, and a change in the time of one behavior leads to changes in the others [7]. Therefore, more and more researchers have changed to investigate the combined effects of PA, SB, and sleep on physical and mental health instead of examining individual behaviors [8–10]. Notably, the existing evidence sheds light on the importance of the combinations of the three behaviors on physical, mental, and cognitive health [8, 11–16]. Several countries have developed national 24-hour movement guidelines based on integrated perspectives [17–20]. However, different terms were used to describe the PA, SB, and sleep collectively in the literature, such as 24-hour movement behaviors [17], physical behaviors [21], time-use activity behaviors [22, 23], and 24-hour activity cycle [24]. To avoid confusion, we adopt the term “24-hour movement behaviors” to collectively describe PA, SB, and sleep in this study.

In order to further understand the 24-hour movement behaviors and their health implications, it is essential to develop validated and reliable instruments to assess 24-hour movement behaviors. In the existing studies of 24-hour movement behaviors, PA, SB, and sleep were either objectively measured by wearable devices (e.g., accelerometers) [25, 26] or subjectively measured by questionnaires and activity logs [27–29]. Although device-based objective measures generally have high validity and reliability [30, 31], the devices are costly, and a high adherence from the participants is required. Meanwhile, the wearable device-based measurements cannot capture the information on the domains of PA and types of SB. Recently, activity log-based methods are also used in some studies to capture the details of PA, SB, and sleep over the 24-hour cycle, such as the Act24 (Activities Completed over Time in 24 h) [32] and STAR24 (Screen Time and Activity Recall) [33]. However, this method is time-consuming and subject to recall bias, and it is not feasible to implement in large-scale epidemiological studies.

In contrast, self-report questionnaires have advantages in reducing the burden on participants and researchers, lower costs, and feasibility [34]. Therefore, questionnaires are still commonly used in health surveillance systems [35]. However, questionnaires for assessing 24-hour movement behaviors are still sparse. Recently, Song and colleagues developed a 24-hour movement behaviors questionnaire for youth in South Korea [36]. It considers the unique lifestyle and environmental characteristics of Korean adolescents (e.g., the college entrance examination-oriented education system). Besides, Kastelic et al. developed the Daily Activity Behaviors Questionnaire (DABQ) for estimating the time spent on sleep, SB, and PA for adults [37]. The DABQ is designed to assess occupational, commuting, and other non-occupational SB and physical activity. Thus, they are not suitable for college students.

The college years are a developmental transition period from late adolescence to emerging adulthood [38]. Promoting college students’ lifestyles and health has attracted great interest worldwide. According to the latest statistics, the number of Chinese college students in 2021 has reached 9.09 million [39]. Therefore, studying 24-hour movement behaviors and their associations with health indicators among college students is important. This information helps create specific lifestyle and health intervention strategies. Unlike in other countries, in China, almost all college students live in dormitories on campus and spend most of their time there. Therefore, developing a specific questionnaire suitable for Chinese college students is necessary.

This study aimed to develop a 24-hour movement behaviors questionnaire for Chinese college students that can be used in large-scale epidemiological studies and examine its reliability and validity.

## Methods

### Participants

This survey was administered to Chinese college students. Convenience sampling was employed to recruit college students via campus advertisements and social networks. Eligible participants were between 18 and 35 years of age, full-time Chinese students, knew the purpose of this study, and voluntarily participated in the cognitive interview or completed the questionnaire. This study has been reviewed and approved by the Ethics Committee of Shanghai Jiao Tong University (approval number: H2022225I).

### Steps of development

**Fig. 1:**
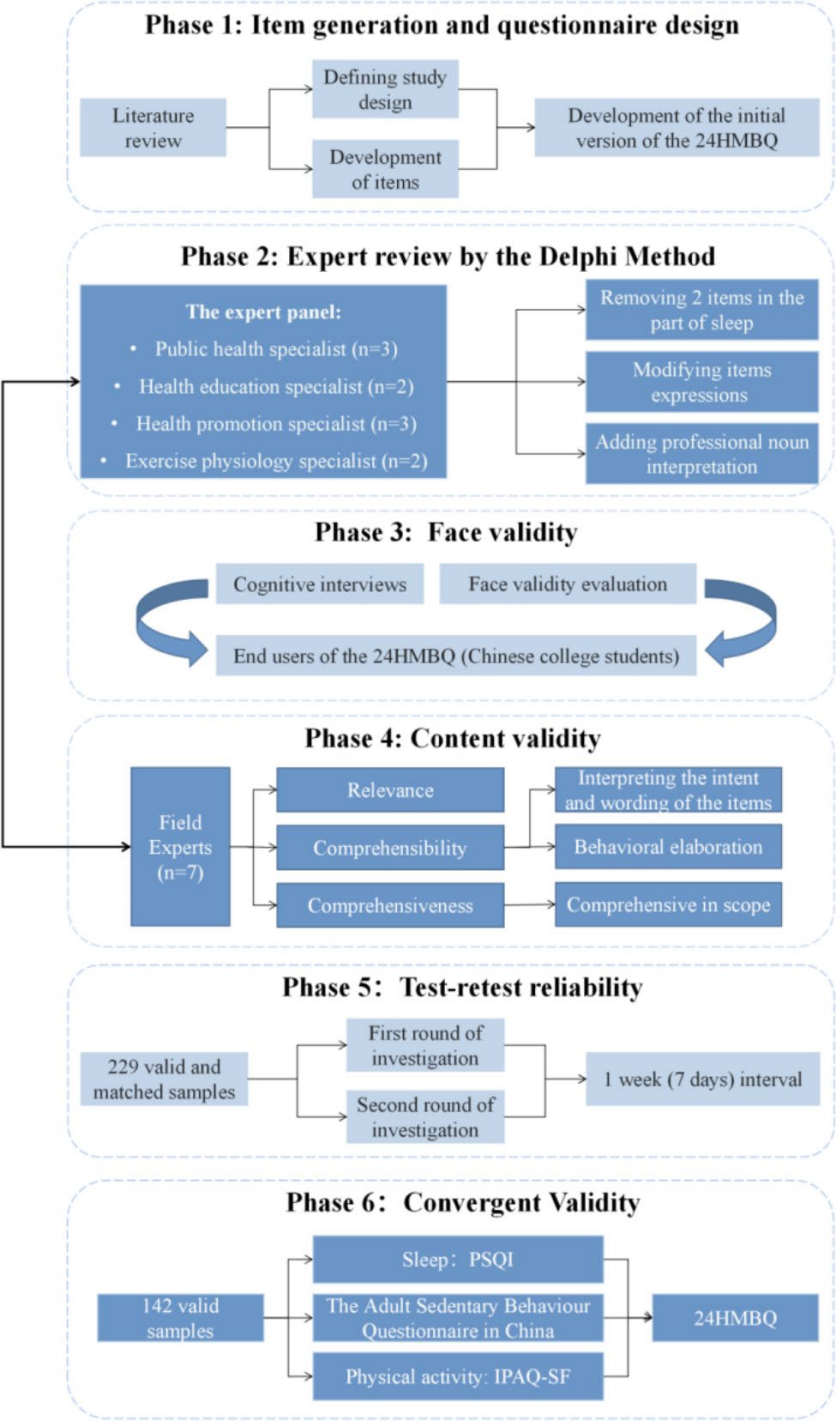
Flow chart of the questionnaire development process

According to the methods of previous studies [21–23], a 24-hour movement behaviors questionnaire (24HMBQ) for Chinese college students was developed through 6 phases (Fig. 1): (1) item generation and questionnaire design; (2) expert review of the initial questionnaire by the Delphi Method; (3) face validity of the revised questionnaire; (4) content validity and final revision of the questionnaire; (5) the test-retest reliability evaluation; and (6) the convergent validity evaluation. Qualitative and quantitative evaluations were used in the questionnaire design process. Figure 1 displays the phases of the questionnaire development process.

### Questionnaire design and expert review (phases 1 and 2)

First, a comprehensive literature review was undertaken. Four electronic databases (PubMed, Web of Science, Google Scholar, and CNKI) were searched to retrieve relevant studies on PA, SB, and sleep. The combination of the following keywords was used: (“physical activity” OR “activity” OR “sedentary behaviors” OR “screen time” OR “sleep” AND “questionnaire”). The following criteria for inclusion were applied: (1) studies should measure PA, SB, and/or sleep; (2) the study should utilize a self-reported questionnaire; and (3) the properties (e.g., reliability and validity) of the questionnaire should be evaluated. The followings were the criteria for exclusion: (1) studies designed to test the effects of certain exercise intervention programs; (2) studies limited to specific demographic categories, such as overweight and/or obese individuals, athletes, or disabled individuals.

Second, the following data were retrieved: (1) the items regarding PA, SB, and sleep; (2) the recall length required for the questionnaire; (3) the different dimensions measurement of PA (e.g., frequency, intensity, and domain); (4) the format of the questionnaire and the number of items; (5) the evaluation methods for reliability and validity; (6) other details of the questionnaire, such as sample size, test-retest interval, data analysis methods, and data comparison measurements, etc.

Items were generated by categorizing and selecting the indicators of 24-hour movement behaviors based on the items of the existing relevant questionnaires. According to the fundamental principles and primary structural requirements of the questionnaire design, the initial version included the demographic information, sleep duration (eight items), duration of different types of SB (six items), the interruption time boundaries of SB (two items), and duration of different domains of PA (nineteen items).

Finally, the initial version of the 24HMBQ was critically evaluated using the Delphi Method. The Delphi Method is considered a reliable measurement instrument for developing new concepts and setting the direction of future-orientated research [40]. This method assesses the extent of agreement and the importance of each issue by seeking the opinions of an expert panel [41]. The expert panel was asked to independently rate all items across three domains using a 5-point Likert scale (“not at all important,” “not important,” “average,” “relatively important,” and “very important”) [42]. The coefficient of variation (CV) indicates the degree of experts’ divergences on the indicator’s importance, the calculation formula’s rationality, and the collection method’s operability. Usually, CV < 0.25 indicates acceptable divergences [41]. Based on the expert panel’s evaluations and suggestions, the initial version of the 24HMBQ was further revised.

### Face validity (phase 3)

Face validity is important for assessing the appropriateness of items in a questionnaire [43, 44]. The target group’s understanding of items is important in deciding face validity. Twenty college students were invited to undergo in-depth interviews. The participants provided their overall impression and understanding of the 24HMBQ and evaluated the content, clarity, and comprehensibility.

Each interview lasted 0.5 to 1 h. The interview content and related information were recorded, and cognitive biases between the researchers and the participants were analyzed. Based on the interviews, each item was analyzed and revised considering wording, comprehension, interpretation, relevance, and coverage.

### Content validity (phase 4)

Content validity is one of the most important attributes of a self-report measurement instrument [45]. It refers to the extent to which the content of a measurement instrument adequately reflects the structure being measured [46]. Seven experts formed a panel to assess the content validity of the 24HMBQ. The experts were requested to evaluate each item’s relevance, clarity, simplicity, and necessity to ascertain its quantitative content validity. Each item was scored on a 4-point Likert scale, from 1 to 4 for “irrelevant” to “highly relevant.” The item-level content validity index (I-CVI), the scale-level content validity index (S-CVI), the mean S-CVI, and the probability of random consistency (Pc) were calculated, and the Pc was corrected by combining the I-CVI to obtain modified Kappa statistic (K*). Several items with a score of 3 or 4/total number of items; mean S-CVI is the mean of I-CVI of all items; Pc = {n! / [A! (n-A)!]}× 0.5^n^, where n is the number of participating experts, and A is the number of experts who gave a rating of 3 or 4; K*=[(I-CVI-Pc)/(1-Pc)] [47].

Content validity is considered good if I-CVI ≥ 0.78 [48]. The unanimous S-CVI should be ≥ 0.80 [49], and the average S-CVI should be ≥ 0.90. The evaluation criteria for K* are 0.40–0.59 for fair, 0.60–0.74 for good, and > 0.74 for excellent [50].

### Reliability (phase 5)

Trained researchers administered the 24HMBQ to the target population to verify its test-retest reliability. The participants were requested to complete the 24HMBQ twice (test-retest validity) at a one-week interval. The survey follows the basic principle of “voluntary, informed, and consent”. The respondents independently filled out the questionnaire.

### Convergent validity (phase 6)

The convergent validity was evaluated using the Pittsburgh Sleep Quality Index (PSQI) [51], the Adult Sedentary Behaviors Questionnaire in China (ASBQC) [52], and the International Physical Activity Questionnaire - Short Form (IPAQ-SF) [53]. The PSQI and IPAQ-SF are often used in isolation to measure sleep and PA, which are acceptably reliable in a Chinese [54, 55]. The Adult Sedentary Behaviors Questionnaire in China is adapted from the Sedentary Behaviors Questionnaire (SBQ) [56].

### Statistical methods

All data analyses were conducted using SPSS (version 25.0, SPSS Inc, 2021). The intra-class correlation coefficient (ICC) was calculated for the test-retest reliability assessment to evaluate the consistency between the first and second administration of the 24HMBQ. Convergent validity was assessed using pairwise non-parametric correlations (Spearman’s) and 95% confidence interval (CI) calculated by bootstrap estimations with 1000 [57]. The criteria for Spearman correlation coefficients were weak (< 0.30), low (0.30–0.49), moderate (0.50–0.69), strong (0.70–0.89), very strong (≥ 0.90) [58]. The criteria for ICC coefficients for test-retest reliability were poor (< 0.50), moderate (0.50–0.74), good (0.75–0.89), and excellent (≥ 0.90) [59]. Mean differences and 95% CI between the first and second administrations of the 24HMBQ were estimated. P < 0.05 was considered to be statistically significant.

## Results

### Expert review and face validity

All the experts have doctoral degrees and have worked in the public health field for ≥ 5 years. Two evaluation rounds were conducted until consensus was achieved among all excepts (Table 1). After expert review, two items were removed due to repetition of other items. On the thirty-three remaining items, panelists reached a reasonable degree of consensus, and modifications were made to make them clearer and more exact. All twenty respondents commented positively on the 24HMBQ, indicating that the item was concise and easy to understand, without ambiguity, and with clear response option categories.

**Table 1:** Result of expert review by the Delphi method

Item	Score		
	Average	SD	CV
During the past week, what time did you usually go to bed at night on weekdays?	4.43	0.79	0.18
During the past week, what time did you usually wake up in the morning on weekdays?	4.43	0.79	0.18
During the past week, how much time did you usually spend sleeping during a day on weekdays?	3.43	2.15	0.63
During the past week, how much time did you usually spend taking a nap during a day on weekdays?	4.14	0.90	0.22
During the past week, what time did you usually go to bed at night on weekends?	4.43	0.79	0.18
During the past week, what time did you usually wake up in the morning on weekends?	4.14	0.90	0.22
During the past week, how much time did you usually spend sleeping during a day on weekends?	4.00	1.91	0.48
During the past week, how much time did you usually spend taking a nap during a day on weekends?	4.14	0.90	0.22
During the past week, on average, how much time per day did you sit to study (including taking courses, self-studying, etc.) or work on weekdays?	4.71	0.49	0.10
During the past week, on average, how much time per day did you spend on electronic screen-based devices for entertainment while sitting or lying on weekdays?	4.86	0.38	0.08
During the past week, on average, how much time per day did you spend on sitting or lying for other sedentary behaviors (e.g. having meals, transportation) on weekdays?	4.29	0.76	0.18
During the past week, on average, how much time per day did you sit to study (including taking courses, self-studying, etc.) or work on weekends?	4.57	0.79	0.17
During the past week, on average, how much time per day did you spend on electronic screen-based devices for entertainment while sitting or lying on weekends?	4.71	0.49	0.10
During the past week, on average, how much time per day did you spend on sitting or lying for other sedentary behaviors (e.g. having meals, transportation) on weekends?	4.14	0.69	0.17
During the past week, on average, how often did you break up sitting during the study or work? (e.g. standing up to relax, going to the tea room)	4.14	0.90	0.22
During the past week, on average, how often did you break up sedentary behaviors during the above-mentioned entertainments using electronic screen-based devices?	4.14	0.90	0.22
During the past week, how often did you do vigorous-intensity physical activity in daily exercise (including workout, PE class, etc.)?	4.86	0.38	0.08
During the past week, how much time did you do vigorous-intensity physical activity in daily exercise (including workout, PE class, etc.)?	4.71	0.49	0.10
During the past week, how often did you do moderate-intensity physical activity in daily exercise (including workout, PE class, etc.)?	4.71	0.49	0.10
During the past week, how much time did you moderate-intensity physical activity in daily exercise (including workout, PE class, etc.)?	4.86	0.38	0.08
During the past week, how often did you do light-intensity physical activity in daily exercise (including workout, PE class, etc.)?	4.86	0.38	0.08
During the past week, how much time did you light-intensity physical activity in daily exercise (including workout, PE class, etc.)?	4.86	0.38	0.08
During the past week, how often did you do vigorous-intensity physical activity in daily transportation?	4.29	0.76	0.18
During the past week, how much time did you do vigorous-intensity physical activity in daily transportation?	4.29	0.76	0.18
During the past week, how often did you do moderate-intensity physical activity in daily transportation?	4.29	0.76	0.18
During the past week, how much time did you do moderate-intensity physical activity in daily transportation?	4.29	0.76	0.18
During the past week, how often did you do light-intensity physical activity in daily transportation?	4.29	0.76	0.18
During the past week, how much time did you do light-intensity physical activity in daily transportation?	4.29	0.76	0.18
During the past week, how often did you do vigorous-intensity physical activity in daily dormitory life?	4.14	0.69	0.17
During the past week, how much time did you do vigorous-intensity physical activity in daily dormitory life?	4.14	0.69	0.17
During the past week, how often did you do moderate-intensity physical activity in daily dormitory life?	4.14	0.90	0.22
During the past week, how much time did you do moderate-intensity physical activity in daily dormitory life?	4.14	0.90	0.22
During the past week, how often did you do light-intensity physical activity in daily dormitory life?	4.86	0.38	0.08
During the past week, how much time did you do light-intensity physical activity in daily dormitory life?	4.86	0.38	0.08
During the past week, how many days did you do mucsle strength training? (e.g. with fitness equipment, bodyweight training such as push-ups)	4.86	0.38	0.08

### Content validity

The I-CVI of most items is greater than 80%, except for the two items of “nap duration on weekdays” and “nap duration on weekends”. Experts rated twenty-nine items with 100% scores, two items with 80% scores, and two items with 70% scores, which are acceptable. The S-CVI was computed at 87.88% and 97.40%, respectively (Table 2). The traditional Chinese view is that naps can supplement the lack of sleep at night and be beneficial to physical and mental health [60]. Considering that day naps are common in China, “nap duration on weekdays” and “nap duration on weekends” are kept following consultation with experts (see Additional file 1 for the English version of the 24HMBQ).

**Table 2:** Content validity for the 24HMBQ

Item	Expert							Experts in agreement	I-CVI		UA	Pc	K*	Evaluation
	Z	Q	C	L	X	Y	F							
Q1	1.00	1.00	1.00	1.00	1.00	1.00	1.00	7.00	1.00		1.00	0.01	1.00	Excellent
Q2	1.00	1.00	1.00	1.00	1.00	1.00	1.00	7.00	1.00		1.00	0.01	1.00	Excellent
Q3	0.00	1.00	1.00	1.00	0.00	1.00	1.00	5.00	0.71		0.00	0.02	0.71	Good
Q4	1.00	1.00	1.00	1.00	1.00	1.00	1.00	7.00	1.00		1.00	0.01	1.00	Excellent
Q5	1.00	1.00	1.00	1.00	1.00	1.00	1.00	7.00	1.00		1.00	0.01	1.00	Excellent
Q6	0.00	1.00	1.00	1.00	0.00	1.00	1.00	5.00	0.71		0.00	0.02	0.71	Good
Q7	1.00	1.00	1.00	1.00	1.00	1.00	1.00	7.00	1.00		1.00	0.01	1.00	Excellent
Q8	1.00	1.00	1.00	1.00	1.00	1.00	1.00	7.00	1.00		1.00	0.01	1.00	Excellent
Q9	1.00	1.00	1.00	1.00	1.00	1.00	1.00	7.00	1.00		1.00	0.01	1.00	Excellent
Q10	1.00	1.00	1.00	1.00	1.00	1.00	1.00	7.00	1.00		1.00	0.01	1.00	Excellent
Q11	1.00	1.00	1.00	1.00	1.00	1.00	1.00	7.00	1.00		1.00	0.01	1.00	Excellent
Q12	1.00	1.00	1.00	1.00	1.00	1.00	1.00	7.00	1.00		1.00	0.01	1.00	Excellent
Q13	1.00	0.00	1.00	1.00	1.00	1.00	1.00	6.00	0.86		0.00	0.01	0.86	Excellent
Q14	1.00	0.00	1.00	1.00	1.00	1.00	1.00	6.00	0.86		0.00	0.01	0.86	Excellent
Q15	1.00	1.00	1.00	1.00	1.00	1.00	1.00	7.00	1.00		1.00	0.01	1.00	Excellent
Q16	1.00	1.00	1.00	1.00	1.00	1.00	1.00	7.00	1.00		1.00	0.01	1.00	Excellent
Q17	1.00	1.00	1.00	1.00	1.00	1.00	1.00	7.00	1.00		1.00	0.01	1.00	Excellent
Q18	1.00	1.00	1.00	1.00	1.00	1.00	1.00	7.00	1.00		1.00	0.01	1.00	Excellent
Q19	1.00	1.00	1.00	1.00	1.00	1.00	1.00	7.00	1.00		1.00	0.01	1.00	Excellent
Q20	1.00	1.00	1.00	1.00	1.00	1.00	1.00	7.00	1.00		1.00	0.01	1.00	Excellent
Q21	1.00	1.00	1.00	1.00	1.00	1.00	1.00	7.00	1.00		1.00	0.01	1.00	Excellent
Q22	1.00	1.00	1.00	1.00	1.00	1.00	1.00	7.00	1.00		1.00	0.01	1.00	Excellent
Q23	1.00	1.00	1.00	1.00	1.00	1.00	1.00	7.00	1.00		1.00	0.01	1.00	Excellent
Q24	1.00	1.00	1.00	1.00	1.00	1.00	1.00	7.00	1.00		1.00	0.01	1.00	Excellent
Q25	1.00	1.00	1.00	1.00	1.00	1.00	1.00	7.00	1.00		1.00	0.01	1.00	Excellent
Q26	1.00	1.00	1.00	1.00	1.00	1.00	1.00	7.00	1.00		1.00	0.01	1.00	Excellent
Q27	1.00	1.00	1.00	1.00	1.00	1.00	1.00	7.00	1.00		1.00	0.01	1.00	Excellent
Q28	1.00	1.00	1.00	1.00	1.00	1.00	1.00	7.00	1.00		1.00	0.01	1.00	Excellent
Q29	1.00	1.00	1.00	1.00	1.00	1.00	1.00	7.00	1.00		1.00	0.01	1.00	Excellent
Q30	1.00	1.00	1.00	1.00	1.00	1.00	1.00	7.00	1.00		1.00	0.01	1.00	Excellent
Q31	1.00	1.00	1.00	1.00	1.00	1.00	1.00	7.00	1.00		1.00	0.01	1.00	Excellent
Q32	1.00	1.00	1.00	1.00	1.00	1.00	1.00	7.00	1.00		1.00	0.01	1.00	Excellent
Q33	1.00	1.00	1.00	1.00	1.00	1.00	1.00	7.00	1.00		1.00	0.01	1.00	Excellent
Proportion relevance									** S-CVI/Ave 0.97**					
	0.94	0.94	1.00	1.00	0.94	1.00	1.00		** S-CVI/UA 0.88**					
The average proportion of items judged as relevant across the seven experts										0.97				

### Reliability

Two hundred forty-eight college students were recruited from China and surveyed twice to evaluate the test-retest reliability. A total of 229 (48.4% male, 51.5% female) college students provided complete data. The mean age of the participants was 21.30 ± 2.52 years.

The ICC for all thirty-three items ranged from 0.68 to 0.97 (Additional file 2), indicating that the test-retest reliability was moderate to excellent. ICC for sleep during the weekday, sleep at the weekend, SB during the weekday, and SB at the weekend were 0.80, 0.74, 0.80, and 0.65, respectively (P < 0.01), indicating good-to-excellent test-retest reliability. ICC for three domains of PA were 0.80, 0.74, and 0.74, respectively (P < 0.01), also indicating good-to-excellent test-retest reliability (Table 3). The results indicate that the questionnaire has high test-retest reliability and good measurement consistency.

**Table 3:** Test-retest reliability for the questionnaire by categories

Variable	Type	Round 1 ^a^	Round 2 ^a^	Rho ^b^	ICC ^c^
**SLP(h/d)** ^**d**^	Weekday	8.61 ± 1.36	8.70 ± 1.39	0.64**	0.80 (0.74–0.84)
	Weekend	9.79 ± 1.77	9.80 ± 1.71	0.65**	0.74 (0.67–0.80)
**SB (h/d)** ^**e**^	Weekday	11.18 ± 3.96	11.11 ± 4.23	0.68**	0.80 (0.74–0.85)
	Weekend	10.57 ± 3.55	10.55 ± 3.99	0.51**	0.65 (0.55–0.73)
**PA (h/d)**^**f**^	Daily exercise (including workout, PE class, etc.)	1.59 ± 1.83	1.60 ± 1.83	0.69**	0.80 (0.74–0.85)
	Daily transportation	0.95 ± 1.05	0.99 ± 1.12	0.63**	0.74 (0.66–0.80)
	Daily dormitory life	0.62 ± 0.68	0.64 ± 0.75	0.73**	0.74 (0.67–0.80)
**Total duration (h/d)** ^**g**^		23.11 ± 3.18	23.19 ± 3.99	0.59**	0.69 (0.59–0.76)

**P < 0.01 for all correlations between test and retest

^a^ Mean ± Standard deviation (SD); ^b^ Spearman’s correlation coefficient (Rho); ^c^ ICC (95% CI); ^d^ Calculated by time to bed and wake-up time; ^e^ Calculated by the sum of any sitting and/or lying down activities; ^f^ Calculated by the sum of duration to do light-intensity physical activity (LPA), moderate-intensity physical activity (MPA)  and vigorous-intensity physical activity (VPA); ^g^ Calculated by the sum of duration of sleep, SB and PA

### Convergent validity

One hundred sixty-one Chinese college students evaluated the convergent validity. A total of 142 (62.7% male, 37.3% female) college students provided complete data. The mean age of the participants was 19.38 ± 2.53 years.

In terms of the convergent validity, correlations were 0.32 for the duration of sleep per day, 0.33 for total time of physical activity per day, and 0.43 for the duration of sedentary behaviors per day (Table 4).

**Table 4:** Convergent validity for the 24HMBQ

Convergent validity	Spearman’s correlation		ICC (95% CI)
	**Rho**	**P**	
**Sleep**	0.32	<0.01	0.40 (0.16–0.57)
**Sedentary behaviors**	0.43	<0.01	0.61 (0.46–0.72)
**Physical activity**	0.33	<0.01	0.57 (0.40–0.69)

## Discussion

This study described the development of a comprehensive questionnaire that could be used to assess the 24-hour movement behaviors of Chinese college students. The development process followed a sequence of standardized steps. Its validity and reliability were examined. The 24HMBQ consists of thirty-three items, which showed suitable face validity, content validity, test-retest reliability, and convergent validity.

The 24HMBQ was developed based on the literature and expert review. Meanwhile, the target population (Chinese college students) and an expert panel of the 24HMBQ were invited to assess the face and content validity. The expert review process offered suggestions; but, no significant changes to items of the 24HMBQ were requested. The result indicated that the researchers and the expert panel consistently understood the items for 24-hour movement behaviors. Regarding content validity, the S-CVI/UA and S-CVI/Ave were 0.88 and 0.97, respectively, confirming that no meaningful activity items were missing and that all included items were relevant.

Similarly, the cognitive interview results did not lead to significant adjustments. However, the participants’ understanding of some items deviated from the original intent of the researchers. The wording of ambiguous items was modified. In general, the 24HMBQ has good face validity and is highly acceptable to the participants.

The test-retest reliability of the 24HMBQ was moderate to excellent (ICC ranging from 0.69 to 0.98) for all items, which is higher than the test-retest reliability of the DABQ (ICC ranging from 0.59 to 0.69) [37]. This result may be because 24HMBQ was designed for Chinese college students’ everyday lifestyle and behavior, thus making the questions more detailed and accurate. The test-retest reliability of PA in daily exercise showed the highest reliability among the three domains, followed by PA in daily dormitory life and PA in daily transportation. This result may be because self-reports of PA rely on the participants’ memory and response tendencies [42]. Existing studies point out that when people are asked to recall frequent daily behaviors (e.g., PA in daily dormitory life or daily transportation), they are less able to recall specific details of many events [61]. Participants who involved in the cognitive interview indicated that sports and exercise were done less frequently and primarily in PE classes, so they could easily recall these. In contrast, the remaining two domains of PA were conducted every day but with unpredictable frequency and duration, making it difficult to recollect them. As a result, the participants must rely on estimation and inference strategies to provide reports. Even so, the retest reliability (0.74–0.80) was still higher than some PA questionnaires, such as Global physical activity questionnaire (GPAQ) [42]. Generally, the 24HMBQ estimations of the time spent in sleep, SB, and PA showed satisfactory test-retest reliability. There is acceptable convergent validity in the duration of sleep, sedentary behaviors, and the total time of physical activity.

To the best of our knowledge, the 24HMBQ is among the first questionnaires with good validity and reliability for Chinese college students to assess movement behaviors over a 24-hour period. A key strength of the 24HMBQ is the rigorous methods with which it was developed. The phases of expert review, cognitive interview, and content validity test were crucial for reducing reporting errors by ensuring that items and instructions were suitably worded for Chinese college students. Meanwhile, the 24HMBQ enables assessments of sleep and SB on weekdays and weekends, the frequency of breaking SB, the duration of engagement in specific SB types (i.e., study, work, electronic screen-based devices for entertainment), different domains of PA (i.e., daily exercise, daily transportation, daily dormitory life), and the frequency of muscle strength training. Therefore, the 24HMBQ can capture more multidimensional information about PA, SB and sleep, which cannot be obtained by objective measurements.

It is practically important to develop the 24HMBQ to provide valuable information about a complete picture of college student’s 24-hour movement behaviors. The questionnaire could be used to investigate the profile of PA, SB and sleep, the joint and independent associations of PA, SB and sleep with physical and mental health indicators in college students. Furthermore, the 24HMBQ is capable to capture the domain-specific physical activity and different types of sedentary behaviors, which can provide more specific recommendations for the development of interventions can promote health-related behaviors.

However, the 24HMBQ is not without limitations. The questionnaire is designed to measure 24-hour movement behaviors among college students. Therefore, the generalization of 24HMBQ to other age groups is limited. In addition, this questionnaire was developed in Chinese context; therefore, it may not be directly used in other countries due to the different culture. Last, although the convergent validity was assessed in the study, the criterion validity should be evaluated by using objective measures of movement behaviors in future studies.

## Conclusion

The 24HMBQ is the first self-reported instrument to comprehensively assess 24-hour movement behaviors for Chinese college students. It is a feasible questionnaire with suitable validity and moderate to excellent test-retest reliability of all items. It is a promising tool to investigate 24-hour movement behaviors for Chinese college students. The 24HMBQ could be a useful and feasible surveillance tool in large-scale epidemiological studies.

## Electronic supplementary material

Below is the link to the electronic supplementary material.

Supplementary Material 1

Supplementary Material 2

## Data Availability

All data supporting this study’s findings are not publicly available due to participants’ confidentiality. However, they are available from the corresponding author upon reasonable request.

## Abbreviations

PA: Physical activity
SB: Sedentary behaviors
DABQ: The Daily Activity Behaviors Questionnaire
CV: Coefficient of variation
I-CVI: The item-level content validity index
S-CVI: The scale-level content validity index
Pc: The probability of random consistency
K*: Modified Kappa statistic
PSQI: The Pittsburgh Sleep Quality Index
ASBQC: The Adult Sedentary Behaviors Questionnaire in China
IPAQ-SF: The International Physical Activity Questionnaire - Short Form
ICC: Intra-class correlation coefficient
CI: Confidence interval
SD: Standard deviation
LPA: Light-intensity physical activity
MPA: Moderate-intensity physical activity
VPA: Vigorous-intensity physical activity.
