# Supplementary material for: Development of a 24-hour movement behaviors questionnaire (24HMBQ) for Chinese college students: validity and reliability testing
Source: BMC Public Health. 2023 Apr 24;23:752. doi: 10.1186/s12889-023-15393-5 (PMC10124027; doi:10.1186/s12889-023-15393-5)
Supplement: Supplementary file 1 — Supplementary Material 1 [file 12889_2023_15393_MOESM1_ESM.docx]

**24-Hour Movement Behaviors Questionnaire (24HMBQ) for Chinese College Students**

PART ONE: SLEEP

Instruction

The following questions are about your sleep during the past 7 days. Please recall and fill in the time (24-hour clock) and length.

|  | Weekday | Weekend |
| --- | --- | --- |
| During the past week, what time did you usually go to bed at night? | __：__ | __：__ |
| During the past week, what time did you usually wake up in the morning? | __：__ | __：__ |
| During the past week, how much time did you usually spend taking a nap during a day? | __hours __min | __hours __min |

PART TWO: SEDENTARY BEHAVIORS

Instruction

The following questions are about your sedentary behaviors during the past 7 days. Please recall and fill in the length and frequency.

|  | Weekday | Weekend |
| --- | --- | --- |
| During the past week, on average, how much time per day did you sit to study (including taking courses, self-studying, etc.) or work? | __hours__ min | __hours __min |
| During the past week, on average, how often did you break up sitting during the study or work? (e.g. standing up to relax, going to the tea room) | ○＜30 min/times  ○ ≥30 min/times＜60 min/times  ○ ≥60 min/times＜90 min/times  ○ ≥90 min/times＜120 min/times  ○ ≥120 min | |
| During the past week, on average, how much time per day did you spend on electronic screen-based devices for entertainment while sitting or lying? (Note: don’t count the time using electronic screen-based devices for study or work, but including watching TV / movies / short videos, video gaming, and using social media, etc.) | __hours __min | __hours __min |
| During the past week, on average, how often did you break up sedentary behavior during the abovementioned entertainments using electronic screen-based devices? | ○＜30 min/times  ○ ≥30 min/times＜60 min/times  ○ ≥60 min/times＜90 min/times  ○ ≥90 min/times＜120 min/times  ○ ≥120 min | |
| During the past week, on average, how much time per day did spend on sitting or lying for other sedentary behaviors? (e.g. having meals, transportation) | __hours __min | __hours __min |

PART THREE: PHYSICAL ACTIVITY

Instruction

The following questions are about your physical activity during the past 7 days. Please recall and fill in the length and frequency.

|  | **Daily exercise (Including workout, PE class, etc.)** | **Daily transportation** | **Daily dormitory life** |
| --- | --- | --- | --- |
| During the past week, how often and how much time did you do vigorous-intensity physical activity?  (hard, significant increases in breathing or heart rate) | __days/week；  __hours __min/day | __days/week；  __hours __min/day  (e.g. fast bicycling) | __days/week；  __hours __min/day  (e.g. carrying heavy objects) |
| During the past week, how often and how much time did you do moderate-intensity physical activity?  (a bit hard, increases in breathing or heart rate) | __days/week；  __hours __min/day | __days/week；  __hours __min/day  (e.g. brisk walking, bicycling) | __days/week；  __hours __min/day  (e.g. carrying light objects, cleaning) |
| During the past week, how often and how much time did you do light-intensity physical activity?  (light, no obvious increases in breathing and heart rate) | __days/week；  __hours __min/day | __days/week；  __hours __min/day  (e.g. skateboarding, walking) | __days/week；  __hours __min/day  (e.g. housework, organizing the closets, folding clothes) |
| **Muscle strength training** | | | |
| During the past week, how many days did you do muscle strength training?  (e.g. with fitness equipment, bodyweight training such as push-ups) | ○ None  __days per week | | |
