# Supplementary material for: Development of a 24-hour movement behaviors questionnaire (24HMBQ) for Chinese college students: validity and reliability testing
Source: BMC Public Health. 2023 Apr 24;23:752. doi: 10.1186/s12889-023-15393-5 (PMC10124027; doi:10.1186/s12889-023-15393-5)
Supplement: Supplementary file 2 — Supplementary Material 2 [file 12889_2023_15393_MOESM2_ESM.docx]

**Additional file 2. Test-retest reliability for the 24HMBQ**

| Variable | Type | Item | Round 1 ^a^ | Round 2 ^a^ | Rho | ICC ^c^ |
| --- | --- | --- | --- | --- | --- | --- |
| Sleep | Weekdays | Q1: During the past week, what time did you usually go to bed at night? ^d^ | 7.68±1.17 | 7.76±1.17 | 0.54** | 0.76  (0.67 - 0.80) |
|  |  | Q2: During the past week, what time did you usually wake up in the morning? ^d^ |  |  |  |  |
|  |  | Q3: During the past week, how much time did you usually spend taking a nap during a day? | 0.93 ± 0.64 | 0.94 ± 0.63 | 0.68** | 0.79  (0.73 - 0.84) |
|  | Weekends | Q4: During the past week, what time did you usually go to bed at night? ^d^ | 8.61 ± 1.35 | 8.63 ± 1.37 | 0.64** | 0.76  (0.69 - 0.81) |
|  |  | Q5: During the past week, what time did you usually wake up in the morning? ^d^ |  |  |  |  |
|  |  | Q6: During the past week, how much time did you usually spend taking a nap during a day? | 1.18 ± 1.00 | 1.17 ± 0.97 | 0.62** | 0.79  (0.73 - 0.84) |
| Sedentary Behaviors | Weekdays | Q7: During the past week, on average, how much time per day did you sit to study (including taking courses, self-studying, etc.) or work? | 5.66 ± 2.75 | 5.57 ± 3.01 | 0.53** | 0.69  (0.60 - 0.76) |
|  |  | Q8: During the past week, on average, how much time per day did you spend on electronic screen-based devices for entertainment while sitting or lying? | 3.51 ± 2.37 | 3.50 ± 2.33 | 0.93** | 0.97  (0.96 - 0.98) |
|  |  | Q9: During the past week, on average, how much time per day did spend on sitting or lying for other sedentary behaviors? (e.g. having meals, transportation) | 2.02 ± 1.47 | 2.04 ± 1.67 | 0.67** | 0.74  (0.66 - 0.80) |
|  | Weekends | Q10: During the past week, on average, how much time per day did you sit to study (including taking courses, self-studying, etc.) or work? | 4.01 ± 2.68 | 4.00 ± 3.01 | 0.51** | 0.70  (0.60 - 0.77) |
|  |  | Q11: During the past week, on average, how much time per day did you spend on electronic screen-based devices for entertainment while sitting or lying? | 4.46 ± 2.58 | 4.46 ± 2.67 | 0.53** | 0.75  (0.67 - 0.80) |
|  |  | Q12: During the past week, on average, how much time per day did spend on sitting or lying for other sedentary behaviors? (e.g. having meals, transportation) | 2.10 ± 1.26 | 2.09 ± 1.32 | 0.65** | 0.68  (0.59 - 0.76) |
|  |  | Q13: During the past week, on average, how often did you break up sitting during the study or work? (e.g. standing up to relax, going to the tea room) ^e^ | 2.37 ± 1.00 | 2.35 ± 1.01 | 0.72** | 0.85  (0.80 - 0.88) |
|  |  | Q14: During the past week, on average, how often did you break up sedentary behavior during the abovementioned entertainments using electronic screen-based devices? ^e^ | 2.45 ± 1.09 | 2.50 ± 1.10 | 0.67** | 0.82  (0.77 - 0.86) |
| Physical Activity | Daily exercise (Including workout, PE class, etc.) | Q15: During the past week, how often did you do physical activity? | 2.43 ± 2.22 | 2.45 ± 2.24 | 0.92** | 0.96  (0.95 - 0.97) |
|  |  | Q16: During the past week, how much time did you do vigorous-intensity physical activity? | 1.10 ± 1.25 | 1.16 ± 1.31 | 0.69** | 0.73  (0.64 - 0.79) |
|  |  | Q17: During the past week, how often did you do moderate-intensity physical activity? | 2.55 ± 2.12 | 2.52 ± 2.19 | 0.69** | 0.81  (0.75 - 0.85) |
|  |  | Q18: During the past week, how much time did you do moderate-intensity physical activity? | 1.05 ± 0.99 | 1.02 ± 1.02 | 0.71** | 0.78  (0.71 - 0.83) |
|  |  | Q19: During the past week, how often did you do light-intensity physical activity? | 2.20 ± 2.29 | 2.21 ± 2.34 | 0.59** | 0.73  (0.65 - 0.79) |
|  |  | Q20: During the past week, how much time did you do light-intensity physical activity? | 0.79 ± 0.91 | 0.80 ± 1.01 | 0.63** | 0.73  (0.66 - 0.80) |
|  | Daily transportation | Q21: During the past week, how often did you do vigorous-intensity physical activity? | 1.04 ± 1.68 | 1.08 ± 1.81 | 0.86** | 0.86  (0.82 - 0.90) |
|  |  | Q22: During the past week, how much time did you do vigorous-intensity physical activity? | 0.35 ± 0.61 | 0.31 ± 0.57 | 0.86** | 0.85  (0.81 - 0.89) |
|  |  | Q23: During the past week, how often did you do moderate-intensity physical activity? | 1.73 ± 2.13 | 1.74 ± 2.17 | 0.57** | 0.68  (0.58 - 0.75) |
|  |  | Q24: During the past week, how much time did you do moderate-intensity physical activity? | 0.56 ± 0.80 | 0.58 ± 0.84 | 0.59** | 0.75  (0.68 - 0.81) |
|  |  | Q25: During the past week, how often did you do light-intensity physical activity? | 3.60 ± 2.65 | 3.56 ± 2.72 | 0.62** | 0.77  (0.70 - 0.82) |
|  |  | Q26: During the past week, how much time did you do light-intensity physical activity? | 0.83 ± 0.84 | 0.86 ± 0.99 | 0.58** | 0.66  (0.56 - 0.74) |
|  | Daily dormitory life | Q27: During the past week, how often did you do vigorous-intensity physical activity? | 1.08 ± 1.62 | 1.10 ± 1.71 | 0.59** | 0.68  (0.58 - 0.75) |
|  |  | Q28: During the past week, how much time did you do vigorous-intensity physical activity? | 0.34 ± 0.66 | 0.35 ± 0.70 | 0.60** | 0.59  (0.47 - 0.69) |
|  |  | Q29: During the past week, how often did you do moderate-intensity physical activity? | 1.64 ± 1.76 | 1.62 ± 1.86 | 0.64** | 0.70  (0.61 - 0.77) |
|  |  | Q30: During the past week, how much time did you do moderate-intensity physical activity? | 0.46 ± 0.56 | 0.44 ± 0.58 | 0.70** | 0.78  (0.72 - 0.83) |
|  |  | Q31: During the past week, how often did you do light-intensity physical activity? | 3.04 ± 2.51 | 3.06 ± 2.55 | 0.65** | 0.77  (0.70 - 0.82) |
|  |  | Q32: During the past week, how much time did you do light-intensity physical activity? | 0.57 ± 0.63 | 0.55 ± 0.56 | 0.73** | 0.76  (0.69 - 0.82) |
|  | Muscle strength training | Q33: During the past week, how many days did you do strength training?  (e.g. with fitness equipment, bodyweight training such as push-ups) | 1.48 ± 1.94 | 1.51 ± 1.94 | 0.61** | 0.66  (0.56 - 0.74) |

**P < 0.01 for all correlations between test and retest.

^a^ Mean ± SD; ^b^ Spearman's correlation coefficient (Rho); ^c^ ICC (95% CI); ^d^ Calculated by time to bed and wake-up time; ^e^ Response options:＜30 min/times, ≥30 min/times＜60 min/times, ≥60 min/times＜min/times, ≥90 min/times＜min/times, ≥120min.
